# Hypoxemia detection and oxygen therapy practices in neonatal and pediatric wards across seven district and referral hospitals in Rwanda

**DOI:** 10.3389/fped.2025.1526779

**Published:** 2025-03-06

**Authors:** Hyacinthe Mushumbamwiza, Harriet H. Webster, Christine Kayitesi, Jasmine Miller, Nang’andu Chizyuka, Felix Musabirema, Alida Ngwije, Brenda Kateera, Sanctus Musafiri, Lisine Tuyisenge, Hamish R. Graham, Felix Lam, Corneille Ntihabose

**Affiliations:** ^1^ Essential Medicines, Clinton Health Access Initiative, Kigali, Rwanda; ^2^ Essential Medicines, Clinton Health Access Initiative, Boston, MA, United States; ^3^ College of Medicine and Health Sciences, University of Rwanda, Kigali, Rwanda; ^4^ Melbourne Children’s Global Health, University of Melbourne, Parkville, VIC, Australia; ^5^ Clinical and Public Health Services, Ministry of Health, Kigali, Rwanda

**Keywords:** hypoxemia, oxygen, pulse oximetry, pneumonia, Rwanda, neonate, pediatric, inpatient

## Abstract

**Background:**

Hypoxemia, characterized by low levels of oxygen in the blood, is a potentially fatal condition that is commonly found in pediatric and neonatal conditions that drive childhood mortality globally. The only treatment is the provision of medical oxygen, yet children in low-income countries frequently are not diagnosed or treated. In Rwanda, it is important to understand the extent to which pediatric and neonatal inpatients are monitored and treated for hypoxemia, in order to guide policy and clinical decision-making.

**Methods:**

This retrospective cohort was undertaken through review of patient clinical case notes in seven hospitals in Rwanda. All patients, up to 14 years of age, admitted to neonatal or pediatric wards in these hospitals within a 3-month period were included in the study. In each facility, trained clinical data collectors used digital survey tools to capture demographic, clinical and outcome data, including pulse oximetry and oxygen use. Neonates were categorized as less than 1 month of age and under-5 s defined between 1 month and 59 months of age, and older children as 5–14 years of age. Our primary outcomes were proportion of admitted children screened with pulse oximetry, and proportion prescribed oxygen when found to be severely hypoxemic, on admission. Our secondary outcomes included hypoxemia prevalence, and other vital signs recorded on admission, oxygen prescription practices, and pulse oximetry screening practices on the day after admission and the day before discharge.

**Results:**

A total of 3,085 neonatal and pediatric patient case notes were included in the analysis. Of these inpatients 86.3% (CI: 95% 85.0–87.4) were screened with pulse oximetry on admission. Of those screened, 18.6% (CI: 95% 17.2–20.1) were documented to have severe hypoxemia (SpO_2_ < 90%). Of 495 patients with documented severe hypoxemia on admission, 48.3% (CI: 95% 44.0–52.6) had an oxygen prescription recorded on admission, reaching 76.0% treated with oxygen across the course of their admission (CI: 95% 72.0–79.5).

**Conclusions:**

Improvements are required in management of hypoxemia in neonates and pediatric inpatients in Rwanda to ensure all patients are screened and those found to be hypoxemic are treated with medical oxygen.

## 1 Introduction

Oxygen therapy is the only treatment for hypoxemia, low levels of oxygen in the blood, a potentially fatal condition. During the COVID-19 pandemic, the number of patients requiring medical oxygen massively increased (1). Oxygen shortages around the world, particularly in LMICs, brought attention to the need for strong oxygen systems (2). Since then, there has been increased investments in oxygen systems across the world (3).

In response to the COVID-19 pandemic, the Rwanda government along with partners implemented several initiatives to improve access to oxygen. These initiatives included installing 31 pressure swing adsorption (PSA) plants to produce oxygen across the country by 2021, conducting trainings to strengthen the capacities of clinicians at hospitals to administer oxygen safely and effectively, and strengthening the national medical insurance system to reimburse hospitals for their use of oxygen (4).

Aside from COVID-19, hypoxemia arises as a consequence of a variety of underlying pathologies, including pneumonia, asthma, sepsis, meningitis and neonatal complications of prematurity (5). Hypoxemia affects acutely ill patients of all ages, but neonates and children experience the highest prevalence of hypoxemia amongst inpatient populations (6). Increased access to oxygen therapy in pediatric wards of hospitals in low-and-middle income countries (LMICs) has been shown to reduce the odds of childhood mortality from pneumonia by 50% (7).

In Rwanda, it is recommended that all inpatients of all ages are screened on admission to measure their blood oxygen with pulse oximetry as part of vital signs monitoring, and all patients with severe hypoxemia should receive oxygen therapy immediately (8). However, there is currently limited published data on the prevalence of hypoxemia or the coverage of pulse oximetry and oxygen therapy practices for neonatal and pediatric patients in Rwanda. Therefore, the objective of this study was to describe the practices of pulse oximetry use and clinical management hypoxemia amongst the neonatal and pediatric inpatient populations in hospital settings in Rwanda. We aimed to contribute to a better understanding of respiratory care amongst this vulnerable group and identify gaps in clinical practices in order to inform policy maker priorities in this area, ultimately to reduce mortality.

## 2 Materials and methods

### 2.1 Key definitions

Hypoxemia is defined as low levels of oxygen in the blood and can be detected through pulse oximetry, a non-invasive method to measure hemoglobin oxygen pulsed saturation (SpO_2_) (9). This measurement can range from 0%–100%, where any SpO_2_ measured at less than 90% is the threshold described by the World Health Organization (WHO) to represent dangerously low oxygen where oxygen therapy will be required in the majority of cases (9). Moderate hypoxemia is defined as SpO_2_ of 90%–93% which might require oxygen therapy in some conditions (9). In this study, we examined neonates which are newborn infants less than 1 month (<28 days) old (10). Pediatric patients are defined in Rwanda as children 1 month up to 14 years old and in some analyses we split these into children 1–59 months (up to 5 years) and older children (5–14 years) due to differential mortality risks in children under 5 years (10). Patient diagnoses were examined, which include clinical diagnoses and other causes of admission such as infection risk, trauma and syndromes. Patients were grouped into those with and without recorded signs of possible respiratory illness, referred to as respiratory symptoms.

### 2.2 Study setting

The study was conducted in neonatal and pediatric inpatient wards across seven hospitals in Rwanda. Rwanda's health care system provides health services through a tiered structure. It consists of community health workers, health posts, health centers, district hospitals, and referral hospitals. Each level provides increasingly complex care, with referral pathways established between them. Our study included two referral hospitals and five district hospitals. We sampled these purposively, aiming to select a range of referral and district hospitals in a range of geographies and aligned with government prioritization of these hospitals to lead the way in future improvements in respiratory care. These study sites covered 25% of referral hospitals (2 of 8), and 12.5% of district hospitals (5 of 40), and hospitals selected were spread geographically across seven different urban and rural districts, across four of the five provinces. As shown in Table 1, the altitude of these hospitals ranged from 1,493 to 2,212 m above sea level. Hospitals had pediatric and neonatal bed capacity ranging from 26 to 57 and 14 to 40 beds, respectively. Additionally, they registered between 960 and 2,150 annual pediatric admissions, and between 850 and 1,570 neonatal annual admissions.

**Table 1:** Characteristics of hospitals in Rwanda selected as study sites (*N* = 7).

Characteristics	Range across district hospitals (*N* = 5)	Range across referral hospitals (*N* = 2)
Pediatric beds	26–44	26–57
Neonatal beds	14–31	35–40
Annual neonatal admissions^a^	849–1,235	1,003–1,573
Annual pediatric admissions^a^	964–2,465	984–2,144
Altitude (meters above sea level)	1,380–2,099	1,586–1,821

^a^ Annual admissions from patient register extraction from October 2021—September 2022.

### 2.3 Study design and data collection

We undertook a retrospective cohort study of patients admitted into the pediatric and neonatal wards of the hospitals between July 1st and September 30th 2021. All patients less than 15 years of age who were admitted to these wards between those dates were eligible for inclusion in the study. Study investigators were selected from clinical staff at each hospital. Investigators were trained to review each patient case note in full and extract key demographic and clinical variables of interest for the study (See Supplementary Annex A for full list). Investigators were equipped with tablet computers and used data collection software Survey CTO to enter the data. The survey tool was available in both Kinyarwanda and English. The data collection underwent an initial piloting stage to improve the validity and reliability. Data collection took place between November 2021—February 2022. The timing of data collection was chosen to ensure that admissions had a final outcome recorded. This study was approved by the Rwanda National Ethics Committee (RNEC).

### 2.4 Analysis

To assess the clinical practice of using pulse oximeters to screen patients for hypoxemia on admission, a binary variable (screened or not screened) was established based whether an SpO_2_ value was recorded in the case notes on the day of admission. Secondary outcomes included other vital signs recorded on admission, and pulse oximetry screening practices on the day after admission and the day before discharge, for patients with data on these dates.

The study also estimated the prevalence of hypoxemia at admission based on the SpO_2_ value, defining a binary variable (hypoxemic or not) where values less than 90% were determined to be severely hypoxemic, and readings of 90%–100% were not (9). Secondary outcomes examined hypoxemia first detected after day of admission, and patients with moderate hypoxemia (SpO_2_ 90%–93%).

For clinical practices on the use of oxygen therapy amongst those who were severely hypoxemic, a binary variable (prescribed oxygen or not) was created if there was any evidence of oxygen prescribed in the treatment plan on the day of admission. Secondary outcomes examined if this cohort were prescribed oxygen in the days after admission, as well as oxygen prescription amongst those without an SpO_2_ reading of <90%.

All outcomes were estimated with 95% confidence intervals. Outcomes were analyzed disaggregated by age group, with neonates were categorized as less than 1 month of age and under-5 s defined between 1 month and 59 months of age, and older children as 5–14 years of age. All the statistical analyses were performed using Stata 17 (11). Patients with missing age, sex or outcome of admission were excluded from the analysis.

### 2.5 Study sample size

The study sample size was calculated to evaluate the prevalence of patients presenting with severe hypoxemia (SpO_2_ < 90%) on admission with reasonable precision. The following formula was used:

$$N=\frac{Z_{1-\alpha }\;*\;p(1-p)}{d^{2}}$$

where:

N = Sample size required

Z_1−*α*_ = 1.96 (the value of the standard normal distribution corresponding to a confidence interval of α = 0.05)

*p* = 0.075 for post-neonatal children and 0.25 for neonatal children (the proportion anticipated to have SpO_2_ < 90%)

d = 0.02 (the desired precision)

The sample size required to power the study using the parameters above was 666 post-neonatal pediatric children and 1,801 neonates. Routine admissions data was used to estimate that three months of admissions would be required to meet the sample size.

## 3 Results

### 3.1 Participant characteristics

**Figure 1:**
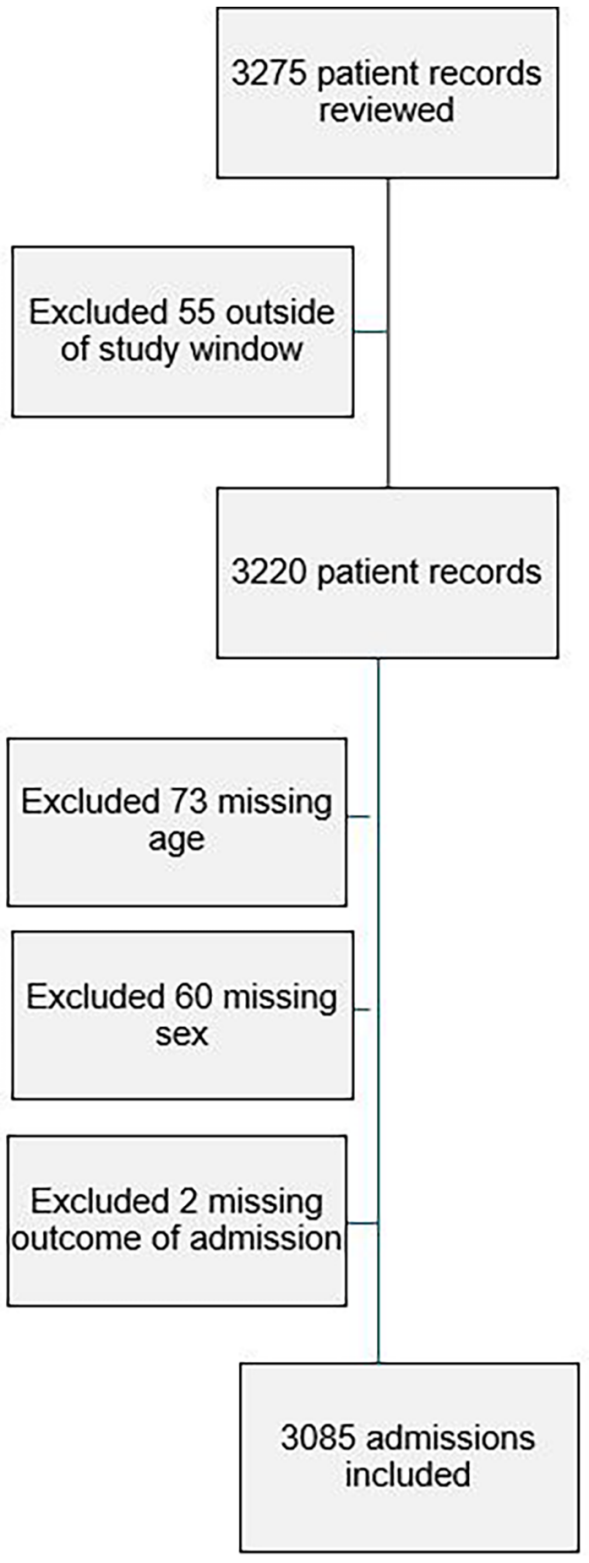
Flowchart of inclusion and exclusion criteria to the study.

Of 3,275 records reviewed, 55 were excluded for being outside the study inclusion dates. A further 73 were excluded for missing age, a further 60 missing sex and further two missing an outcome of admission. A total of 3,085 patient charts were included in the study (Figure 1).

Of these, 48.8%, (*N* = 1,505/3,085, CI: 95% 47.1–50.5%) were neonates (<1 month), 40.8% (*N* = 1,258/3,085, CI: 95% 39.1–42.5%) were under-5 pediatric patients (between 1 month and 4 years), and 10.4% (*N* = 322/3,085, CI: 95% 9.4–11.6%) were older children (5–14 years). Under half, 43.5% (*N* = 1,341/3,085, CI: 95% 41.7–45.2%), of patients were female. The most common length of admission was 3 days (IQR 2–6 days). The majority, 91.9% (*N* = 2,836/3,085, CI: 95% 90.9–92.8%), of patients were discharged at the end of admission, whilst 4.9% (*N* = 150/3,085, CI: 95% 4.2%–5.7%) of patients died. The most common diagnoses on admission for neonates were neonatal infection (risk), prematurity and respiratory distress syndrome. The most common diagnoses on admission for pediatric inpatients aged 1 month up to 14 years were malaria, pneumonia and abdominal/gastrointestinal diagnoses (Table 2).

**Table 2:** Baseline characteristics of inpatients included in the study by month (*N* = 3,085).

Category	July 2021	August 2021	September 2021	Total
Total	963 (31.2)	1,209 (39.2)	913 (29.6)	3,085 (100.0)
Age				
Neonate <1 month	502 (52.1)	562 (46.5)	441 (48.3)	1,505 (48.8)
Child 1–59 months	369 (38.3)	507 (41.9)	382 (41.8)	1,258 (40.8)
Child 5–14 years	92 (9.6)	140 (11.6)	90 (9.9)	322 (10.4)
Sex				
Male	546 (56.7)	689 (57)	509 (55.8)	1,744 (56.5)
Female	417 (43.3)	520 (43)	404 (44.2)	1,341 (43.5)
Respiratory symptoms^a^				
No	575 (59.7)	896 (74.1)	685 (75)	2,156 (69.9)
Yes	388 (40.3)	313 (25.9)	228 (25)	929 (30.1)
Median days of admission (IQR)	4 (2–6)	3 (2–6)	3 (2–5)	3 (2–6)
Admitted From				
Community	338 (35.1)	414 (34.2)	264 (28.9)	1,016 (32.9)
Born at facility	383 (39.8)	451 (37.3)	339 (37.1)	1,173 (38)
Referred from Primary Facility	235 (24.4)	342 (28.3)	308 (33.7)	885 (28.7)
Referred from Higher Facility/Other	7 (0.7)	2 (0.2)	2 (0.2)	11 (0.4)
Outcome				
Discharged alive	872 (90.6)	1,121 (92.7)	843 (92.3)	2,836 (91.9)
Left Against Medical Advice	22 (2.3)	27 (2.2)	23 (2.5)	72 (2.3)
Died	57 (5.9)	52 (4.3)	41 (4.5)	150 (4.9)
Referred out	12 (1.2)	9 (0.7)	6 (0.7)	27 (0.9)
Neonatal diagnoses on admission (<1 month)^b^				
Neonatal Infection (Risk)	149 (28.4)	169 (31.0)	149 (35.6)	467 (31.4)
Prematurity	90 (17.1)	113 (20.7)	74 (17.7)	277 (18.6)
Respiratory Distress Syndrome	69 (13.1)	66 (12.1)	44 (10.5)	179 (12.0)
Birth asphyxia	72 (13.7)	49 (9.0)	39 (9.3)	160 (10.8)
Jaundice	21 (4.0)	32 (5.9)	19 (4.5)	72 (4.8)
Hypoglycemia	28 (5.3)	16 (2.9)	16 (3.8)	60 (4.0)
Septicemia	17 (3.2)	18 (3.3)	17 (4.1)	52 (3.5)
Encephalopathies	15 (2.9)	16 (2.9)	18 (4.3)	49 (3.3)
Respiratory Tract Infection	13 (2.5)	18 (3.3)	8 (1.9)	39 (2.6)
Pneumonia	9 (1.7)	10 (1.8)	4 (1.0)	23 (1.5)
Pediatric diagnoses on admission (1 month—14 years)^b^				
Malaria	88 (22.1)	125 (20.8)	67 (15.9)	280 (19.7)
Pneumonia	87 (21.8)	101 (16.8)	71 (16.9)	259 (18.2)
Abdominal/Gastrointestinal	47 (11.8)	103 (17.1)	87 (20.7)	237 (16.7)
Diarrhea	39 (9.8)	80 (13.3)	64 (15.2)	183 (12.9)
Septicemia	25 (6.3)	41 (6.8)	25 (5.9)	91 (6.4)
Respiratory Tract Infection	31 (7.8)	28 (4.7)	27 (6.4)	86 (6.1)
Seizures/Convulsions	19 (4.8)	28 (4.7)	30 (7.1)	77 (5.4)
COVID-19	13 (3.3)	24 (4)	9 (2.1)	46 (3.2)
Urinary Tract Infection	12 (3.0)	15 (2.5)	10 (2.4)	37 (2.6)
Malnutrition	8 (2.0)	16 (2.7)	6 (1.4)	30 (2.1)

^a^ Respiratory symptoms include cold, cough, crackles, cyanosis, difficulty breathing, fast breathing, grunting, chest indrawing, nasal flaring, stridor, wheezing, birth asphyxia.

^b^ Patients can have multiple diagnoses. The 10 most common diagnoses are shown, see Supplementary Annex B for full table.

### 3.2 Pulse oximetry use practices

Of all 3,085 pediatric and neonatal patients, 86.3% were screened with pulse oximetry on admission (*N* = 2,662/3,085, CI: 95% 85.0–87.4). As shown in Table 3, pulse oximetry screening was similar between patients with respiratory symptoms (84.8%. *N* = 788/929, CI: 95% 82.4–87.0%) and patients without respiratory symptoms (86.9%, *N* = 1,874/2,156, CI: 95% 85.4–88.3%).

**Table 3:** Proportion of inpatients screened with pulse oximetry on day of admission to study sites (*N* = 3,085).

Category	Patients screened with pulse oximetry on day of admission	Total patients (*N*)	% screened (CI: 95%)
Total	2,662	3,085	86.3 (85.0–87.4)
Age			
Neonate <1 month	1,405	1,505	93.4 (92.0–94.5)
Child 1–59 months	1,015	1,258	80.7 (78.5–82.7)
Child 5–14 years	242	322	75.2 (70.2–79.5)
Sex			
Male	1,508	1,744	86.5 (84.8–88.0)
Female	1,154	1,341	86.1 (84.1–87.8)
Respiratory symptoms^a^			
No	1,874	2,156	86.9 (85.4–88.3)
Yes	788	929	84.8 (82.4–87.0)
Admitted from			
Community	813	1,016	80.0 (77.5–82.3)
Born at facility	1,087	1,173	92.7 (91.0–94.0)
Referred from Primary Facility	755	885	85.3 (82.9–87.5)
Referred from Higher Facility Other	7	11	63.6 (33.9–85.7)
Outcome			
Discharged alive	2,457	2,836	86.6 (85.4–87.8)
Run-Away	50	72	69.4 (58.0–78.9)
Died	132	150	88.0 (81.8–92.3)
Referred out	23	27	85.2 (66.5–94.3)

^a^ Respiratory symptoms include cold, cough, crackles, cyanosis, difficulty breathing, fast breathing, grunting, chest indrawing, nasal flaring, stridor, wheezing, birth asphyxia.

Pulse oximetry screening was less common in older age groups, with 93.4% (*N* = 1,405/1,505, CI: 95% 92.0–94.5%) of neonates, less than 1 month old, being screened compared to 75.2% of older children between 5 and 14 years (*N* = 242/322, CI: 95% 70.2–79.5%).

When comparing to other vital signs measurements it was found temperature was most commonly documented on admission, in 93.2% (*N* = 2,876/3,085, CI: 95% 92.3–94.0%) of patients. Respiratory rate was the least documented, in 74.2% (*N* = 2,289/3,085, CI: 95% 72.6–75.7%) of patients, less than pulse oximetry. Pulse rate was documented in 82.4% (*N* = 2,543/3,085, CI: 95% 81.1–83.7%) of patients on admission.

**Figure 2:**
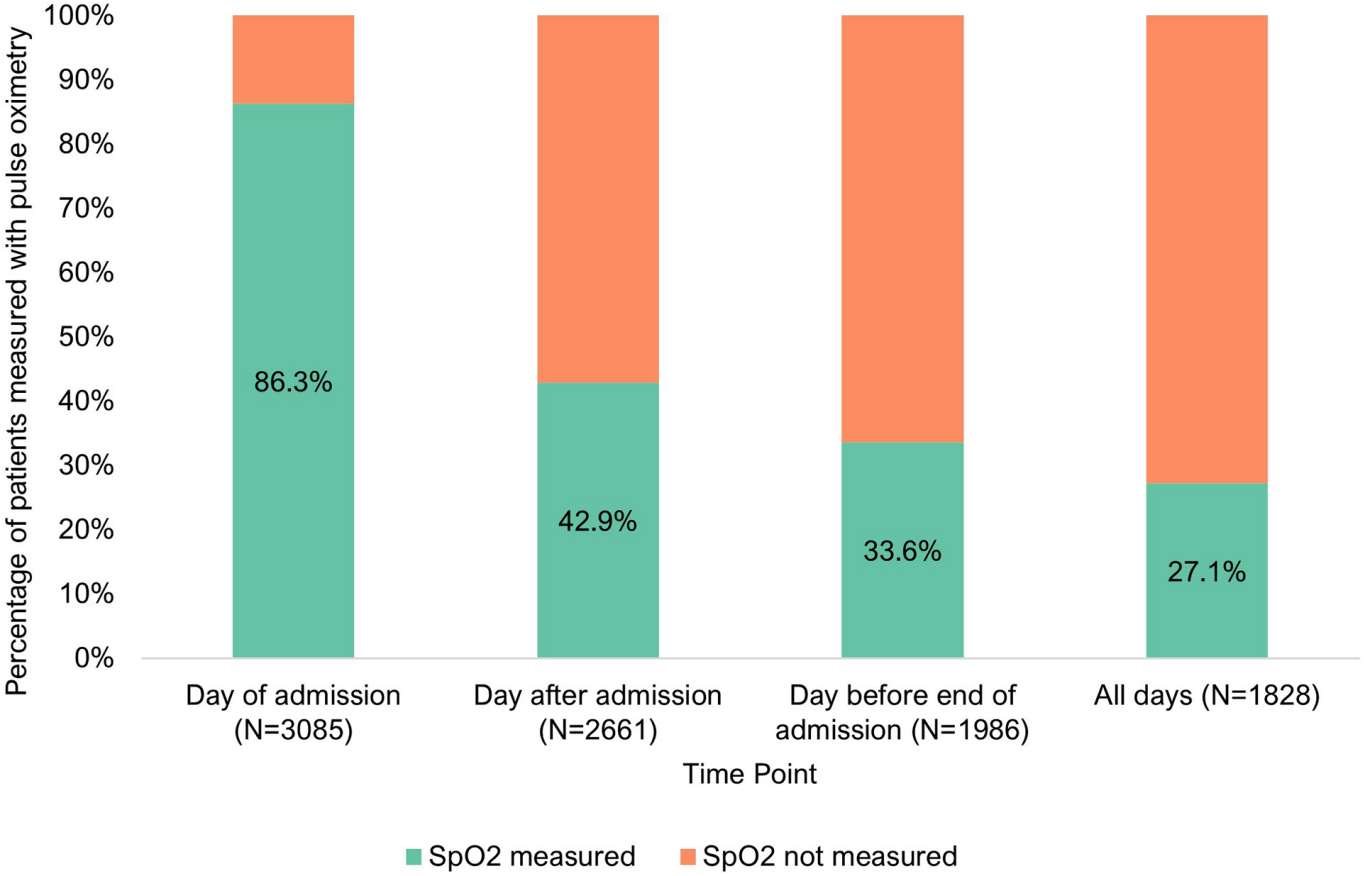
Pulse oximetry monitoring of inpatients at three points of admission [day of admission (*N* = 3,085), day after admission (*N* = 2,661) and day before end of admission (*N* = 1,986)].

Pulse oximetry measurements declined after admission, shown in Figure 2. It was found of 2,661 patients with case notes on the day after admission, 42.9% (*N* = 1,142/2,661, CI: 95% 41.2–44.7%) had pulse oximetry documented. Of 1,986 patients with case notes the day before end of admission, just 33.6% (*N* = 668/1,986, CI: 95% 31.7–35.6%) had pulse oximetry documented.

### 3.3 Hypoxemia prevalence

Of the 2,662 patients that were screened with pulse oximetry on admission, 18.6% were found to be severely hypoxemic (*N* = 495/2,662, CI: 95% 17.2–20.1%). In neonates, 22.8% of those with documented SpO_2_ were severely hypoxemic on admission (*N* = 321/1,405, CI: 95% 20.8–25.1%), 15.2% of children less than five years (*N* = 154/1,015, CI: 95% 13.2–17.5%) and 8.3% of older children (*N* = 20/242, CI: 95% 5.4–12.5%). As shown in Table 4, older children between 5 and 14 years of age were less likely to be severely hypoxemic.

**Table 4:** Proportion of inpatients screened with pulse oximetry on admission to study sites that were found to be hypoxemic (*N* = 2,662).

Category	Patients screened with pulse oximetry on admission found to be hypoxemic	Total Patients screened with pulse oximetry (*N*)	Patients screened with pulse oximetry on admission found to be hypoxemic (CI: 95%)
Total	495	2,662	18.6 (17.2–20.1)
Age Group			
Neonates (<1 month)	321	1,405	22.8 (20.8–25.1)
Under-5 (1–59 months)	154	1,015	15.2 (13.2–17.5)
Older children (5–14 years)	20	242	8.3 (5.4–12.5)
Neonatal Diagnoses^a^ (<1 month)			
Urinary Tract Infection	2	2	100.0
Apnea	4	7	57.1
Pneumonia	12	23	52.2
Respiratory Distress Syndrome	72	171	42.1
Birth asphyxia	58	148	39.2
Encephalopathies	18	49	36.7
Aspiration	6	18	33.3
Malaria	2	6	33.3
Malnutrition	1	3	33.3
Sickle Cell Anemia	1	3	33.3
Seizures/Convulsions	4	14	28.6
Respiratory Tract Infection	11	39	28.2
Prematurity	72	264	27.3
Abdominal/Gastrointestinal	4	18	22.2
Septicemia	10	46	21.7
Hypoglycemia	11	59	18.6
Congenital Malformations	4	22	18.2
Neonatal Infection (Risk)	82	467	17.6
Anemia	1	7	14.3
Jaundice	6	70	8.6
COVID-19	0	4	0.0
Typhoid	0	2	0.0
Diarrhea	0	1	0.0
Tuberculosis	0	1	0.0
Pediatric Diagnoses^a^ (1 month—14 years)			
Asthma	6	19	31.6
Pneumonia	66	211	31.3
Sickle Cell Anemia	2	14	14.3
Malnutrition	3	23	13.0
Trauma	1	8	12.5
Respiratory Tract Infection	8	82	9.8
COVID-19	4	42	9.5
Encephalopathies	1	11	9.1
Meningitis	1	13	7.7
Malaria	19	248	7.7
Diarrhea	8	152	5.3
Seizures/Convulsions	4	77	5.2
Abdominal/Gastrointestinal	11	237	4.6
Septicemia	4	89	4.5
Urinary Tract Infection	1	34	2.9
Typhoid	0	17	0.0
Tuberculosis	0	3	0.0
Otis Media	0	2	0.0

^a^ Patients can have multiple diagnoses. Excluding those with other miscellaneous or no diagnoses.

Of neonates (<1 month) assessed with pulse oximetry, the diagnoses where severe hypoxemia was most prevalent was in urinary tract infections (100%, 2 of 2), apnea (57.1%, 4 of 7) and pneumonia (52.2%, 12 of 23). In pediatric cases (1 month—15 years) assessed with pulse oximetry, the diagnoses where severe hypoxemia was most prevalent was in asthma (31.6%, 6 of 19), pneumonia (31.3%, 66 of 211) and sickle cell anemia (14.3%, 2 of 14). Patients with severe hypoxemia without documented respiratory symptoms were most likely to have diagnosis of being premature.

Of the 2,662 patients screened with pulse oximetry on admission, a further 13.6% were found to be moderately hypoxemic, with an SpO_2_ of 90%–93% (*N* = 361/2,662, CI: 95% 12.3–14.9%).

Of the 1,091 patients screened on admission and the day after, an additional 3.4% (*N* = 29/1,091, CI: 95% 2.4%–4.8%) were found to develop severe hypoxemia the day after admission. Of the 639 patients screened on the day before discharge and the day of admission, an additional 3.6% (*N* = 18/639, CI: 95% 2.3%–5.6%) were found to be severely hypoxemic when they were not on admission.

### 3.4 Oxygen therapy practices

Of the 495 patients with documented severe hypoxemia on admission, 48.3% (*N* = 239/495, CI: 95% 44.0–52.6%) had an oxygen prescription recorded on the same day (Table 5). Neonates were the least likely to receive oxygen on day of admission, with only 39.3% receiving oxygen therapy at that time (*N* = 126/321, CI: 95% 34.2–44.6%) compared to 66.9% of children under 5 (*N* = 103/154, CI: 95% 59.2–73.7%) and 50.0% of older children (*N* = 10/20, CI: 95% 29.4–70.6%). A greater proportion, 75.6%, of these 495 patients with severe hypoxemia on admission were prescribed oxygen at a later point during the admission (*N* = 376/495, CI: 95% 72.0–79.5).

**Table 5:** Provision of oxygen therapy to inpatients diagnosed with hypoxemia on admission to study sites (*N* = 495).

Category	Hypoxemic patients on admission (*N*)	Hypoxemic patients prescribed oxygen on admission	
		*n*	% (CI: 95%)
Total	495	239	48.3 (44.0–52.6)
Sex			
Male	275	121	44.0 (38.4–49.8)
Female	220	118	53.6 (47.1–60.1)
Age group			
Neonate <1 month	321	126	39.3 (34.2–44.6)
Child 1–59 months	154	103	66.9 (59.2–73.7)
Child 5–14 years	20	10	50.0 (29.4–70.6)
Referred prior to admission			
Community	120	70	58.3 (49.7–66.5)
Neonatal born at facility	244	88	36.1 (30.4–42.2)
Referred from primary facility	131	81	61.8 (53.3–69.7)
Symptoms^a^			
No respiratory symptoms	235	92	39.1 (33.2–45.5)
Respiratory symptoms	260	147	56.5 (50.6–62.3)
Outcome			
Discharged alive	409	192	46.9 (42.3–51.7)
Died	72	38	52.8 (42.4–63.9)
Run-Away	11	7	63.6 (33.8–85.7)
Referred out	3	2	66.7 (15.3–95.7)

^a^ Respiratory symptoms include cold, cough, crackles, cyanosis, difficulty breathing, fast breathing, grunting, chest indrawing, nasal flaring, stridor, wheezing, birth asphyxia.

Patients with severe hypoxemia were more likely to be prescribed oxygen on admission, if they were admitted with respiratory symptoms, with 56.5% being prescribed (*N* = 147/260, CI: 95% 56.5–62.3%). Only 39.1% of patients with severe hypoxemia without documented respiratory symptoms (*N* = 92/235, CI: 95% 33.2–45.5%) were prescribed oxygen.

Overall, 593 patients were prescribed oxygen on admission, and of these only 40.3% were severely hypoxemic with SpO_2_ of less than 90% (239/593). Of the remaining patients prescribed oxygen, 15.0% had moderate hypoxemia of SpO_2_ of 90%–93% (68/593), and 36.1% had SpO_2_ of 94%–100% (214/593). The remaining 8.6% were prescribed oxygen without a pulse oximetry measurement documented (51/593). Overall, the most common symptoms amongst the 354 patients who were prescribed oxygen on admission without severe hypoxemia, included prematurity (16.2%, 93/571 symptoms), difficulty breathing (10.3%, 59/571 symptoms), cough (9.5%, 54/571 symptoms) and fever (9.1%, 52/571 symptoms).

There were a reported 935 patients prescribed oxygen at any point during admission. Of these, 822 had a documented flowrate in liters per minute (LPM), this ranged from 0.75 L/min to 10 L/min, with the median being 2l/min (IQR 1–3 LPM). This flowrate information was missing from 16.6% of the prescriptions (155 of 935).

## 4 Discussion

Among 3,085 neonatal and pediatric patients admitted to hospitals in Rwanda, we found 86.3% (CI: 95% 85.0–87.4%) had documented pulse oximetry on admission. Of the 2,662 patients that were screened with pulse oximetry on admission, 18.6% (CI: 95% 17.2–20.1%) had severe hypoxemia (SpO_2_ < 90%). Of 495 patients with documented severe hypoxemia on admission, 48.3% had an oxygen prescription recorded on the same day (CI: 95% 44.0–52.6%).

National clinical guidelines in Rwanda recommend all neonates and pediatric inpatients should be screened with pulse oximetry on admission to hospital (12). From this study we see that in 2021 there was moderately high usage of pulse oximetry on admission in neonatal and pediatric inpatient populations, yet there is still progress to be made to ensure all are measured. Particularly, we found pulse oximetry use dropped significantly after the day of admission, Rwandan national clinical guidelines stipulate pulse oximetry should be carried out every 8 h alongside other vital signs (12). This difference seen in practice may be caused by lack of pulse oximeter devices that are prioritized to routes of admission, or lack of clinical awareness (13). Additionally, recording of respiratory rate, another vital sign, was found to be low. It is important pulse oximetry is used in conjunction with respiratory rate, for a comprehensive clinical diagnosis (14). This should be an area of quality improvement to ensure neonatal and pediatric patients whose condition deteriorates are not missed, and that oxygen therapy dosage and duration is guided by SpO_2_ measurements. Capacity building of healthcare staff, specifically practical training and strong clinical leadership has been shown to improve clinical practices in hypoxemia management (13).

Hypoxemia prevalence estimates found in our study are similar to other studies, particularly prevalence of 22.8% in neonates, which aligned with range of 19.7% to 23.1% in this meta-analysis (5). However, with other illnesses there is a higher degree of heterogeneity between different studies, for example between 6.3% to 100% for very severe pneumonia (5), and 8% for non-severe pneumonia (15). This underscores the importance of understanding local prevalence hypoxemia in patients presenting to care depending on disease epidemiology and care seeking (16).

Oxygen therapy is recommended for all children with SpO_2_ less than 90% and should be prescribed immediately to treat this medical emergency (9). It has been demonstrated SpO_2_ < 90% is strongly associated with an increased risk of death in children with lower-respiratory infections in low- and middle-income countries (17), alongside other risk factors such as severe acute malnutrition (18). Yet we found prescription practices for this group was low on admission in all age groups. Neonates had the highest prevalence of hypoxemia on admission but were least likely to be prescribed oxygen that same day. Prescription to patients found to be hypoxemic on admission increased to 76.0% (376 of 495, CI: 95% 72.0–79.5) when considering the days following admission, suggesting delays in oxygen therapy, however documentation practices not capturing this treatment on admission can't be ruled out. Further investigation is needed to examine how patients with hypoxemia are identified and treated, in order to ensure oxygen is prescribed when required.

Conversely of all patients prescribed oxygen on admission, many had SpO_2_ of 90% or greater. This included 36.1% patients receiving oxygen having an SpO_2_ of 94%–100%. Oxygen therapy may be clinically indicated for patients with higher SpO_2_ values than 89% in specific clinical situations such as during anesthesia and trauma, and the WHO recommends the provision of oxygen to pediatric patients with SpO_2_ < 94% if they also have emergency signs (19). However, particularly pre-term neonates, there is a risk of toxicity resulting in retinopathy of prematurity (20) and chronic lung disease (21). Risk of toxicity might be a factor in the low prescription practices seen in neonates, in some cases. Further exploration into the clinical training of clinicians is required to ensure they have the knowledge to guide oxygen prescription practices as well as ensuring they are equipped with functional accessories and equipment to manage this condition.

Flowrates, the amount of oxygen delivered per minute, should largely follow guidelines on optimal flows for the age group and delivery method (12). This aspect of the prescription was missing in 16.6% of prescription, yet medical oxygen as a drug should not be prescribed without a dose. Further training and enhanced clinical recording systems should optimize patient outcomes by ensuring prescribing aligns with national clinical guidelines (12).

These results align with findings from intervention-based research in a Rwandan tertiary hospital adult emergency unit which found clinical knowledge gaps in understanding which patients require medical oxygen (22). Additionally, quality respiratory care through the detection of hypoxemia and provision of oxygen has recently been highlighted in a needs assessment for neonatal mortality reduction in Rwanda as two of the six key recommendations to improve neonatal outcomes (23).

Given these findings, further clinical training, mentorship and guidelines will ensure clinicians are equipped with comprehensive, current knowledge of best practices to ensure oxygen therapy is clinically required and safe.

### 4.1 Strengths and limitations of the study

This study examines hypoxemia detection, prevalence and treatment in children, and can help guide the government and other stakeholders to strengthen the healthcare system in this area.

The study sample of facilities was purposively chosen and therefore whilst there was an aim for geographic representation, there is a limited ability to generalize to the national level. However the findings provide a guideline for future data collection into this area of clinical practice. Given variation in clinical practice, further research should explore factors that affect use of pulse oximetry and oxygen, in order to better target clinical quality improvement.

The recent time period was chosen to minimize loss of records, however being limited to three months misses the seasonal nature of hypoxemia-related illnesses like pneumonia (24) and therefore caution must be taken in extrapolating findings to understand annual prevalences of hypoxemia.

One key limitation of ascertaining patient care from records is that poor documentation practices may have led to an underestimation of pulse oximetry or oxygen prescription due to incomplete record keeping. Particularly the measure of oxygen prescription to hypoxemic on admission requires further investigation to determine true prescription practices.

Using data collectors with a clinical background ensured greater ability to understand the source data. Good documentation is accepted as a critical part of clinical care, enabling continuity of care and early recognition of deteriorating patients. Documentation requires strong data systems in place with multiple staff cadres working together to capture patient care, from diagnosis, prescription to delivery. This investment benefits patient care as well as providing insights to decision-makers at a ward, hospital and national levels.

## 5 Conclusion

In Rwanda, significant progress has been made in reducing the under 5 mortality rate from 196 to 45 deaths per 1,000 live births between 2000 and 2020. For neonates this reduced from 44 to 19 deaths per 1,000 live births in the same timeframe (25). However, in recent years the decreases have been shallower, particularly amongst neonates. Future research will be required to understand drivers of quality oxygen care clinical settings, both the strengths and gaps in the respiratory care in this setting.

In conclusion, whilst pulse oximetry use in neonatal and pediatric wards in Rwanda is pervasive, there are still children missing out on this vital diagnostic tool and improvements are required to ensure its' use extends beyond the day of admission. Hypoxemia prevalence amongst inpatient children and neonates is high, yet gaps remain in provision of oxygen therapy, particularly on admission. Continued efforts will be required to understand and address these gaps, in order to save more lives in Rwanda.

## Data Availability

The raw data supporting the conclusions of this article will be made available by the authors, without undue reservation.
