# Supplementary material for: Hypoxemia detection and oxygen therapy practices in neonatal and pediatric wards across seven district and referral hospitals in Rwanda
Source: Front Pediatr. 2025 Mar 6;13:1526779. doi: 10.3389/fped.2025.1526779 (PMC11922911; doi:10.3389/fped.2025.1526779)
Supplement: Supplementary file 1 [file Datasheet1.docx]

**Supplementary Annex A: Survey tool used to extract data from patient case notes**

| **SECTION I: IDENTIFICATION** | | | |
| --- | --- | --- | --- |
| **NO.** | **QUESTION** | **RESPONSE CODE** | **SKIP** |
|  | Region |  |  |
|  | Facility type |  |  |
|  | Facility name | (Filtered based on ID1 and ID2) |  |

| **NO.** | **QUESTION** | **RESPONSE CODE** | **SKIP** |
| --- | --- | --- | --- |
|  | Date of admission  Special codes:  *99 = Information missing* | DD/MM/YYYY ____ / ____ / ____ |  |
|  | Date of discharge  Special codes:  *99 = Information missing* | DD/MM/YYYY ____ / ____ / ____ |  |
|  | Discharge status | Discharged alive 1  Referred out 2  Died 3  Other outcome 4  Information missing 9 |  |
|  | Child date of birth (DD/MM/YYYY)  Special codes:  99 = Information missing | DOB ___ ___ / ___ ___ / ___ ___ ___ ___ |  |
|  | Age of the child in years  Enter the age in completed years. For example, a child that is 1 year and 3 months old should be entered as ‘1’. A child that is less than 1 year old should be entered as ‘0’.  Special codes:  0 = child is less than 1 year old  99 = Information missing | Age in completed years ___ ___ |  |
|  | If PC5 is ‘0’:  Age of the child in months  Special codes:  0 = child is less than 1 month old  99 = Information missing | Age in months ___ ___ |  |
|  | Sex of the child | Male 1  Female 2  Information missing 9 |  |
| **Instructions:** You will first collect information about the initial assessment, patient history, and treatment on the first day the child was seen at the facility (Day 0). Do not include information from other days. | | | |
|  | Initial chief complaints and symptoms at admission (Day 0)  Mark all that apply. | **Respiratory**  Chest in-drawing R01  Cold R02  Cough R03  Crackles R04  Cyanosis/blue discoloration of the skin R05  Difficulty breathing R06  Fast breathing/Tachypnea R07  Grunting sound R08  Hypoxemia (low oxygen saturation) R09  Nasal flaring R10  Stridor (wheezing/grating sound) when  child is calm R11  Wheezing sound R12  Bradypnea R13  **Gastro/Feeding**  Diarrhoea F1  Unable to eat normally F2  Unable to drink normally F3  Vomiting F4  Nausea F5  Abdominal pain F6  **Neuro**  Convulsions N1  Drowsy or difficult to rouse N2  Pain (including headache and joint pain) N3  Restlessness or easily irritable N4  Unable to sleep normally N5  Unconscious N6  **General**  Fatigue or no energy G1  Fever G2  Shivering or chills G3  Dehydration G4  Other (*specify*) X  None (no symptoms recorded) Z |  |
|  | Number of days the patient was ill before being admitted  Special codes:  0 = Same day as illness began  99 = Information missing | Number of days ___ ___ |  |
|  | Child’s weight in kg at admission (Day 0)  Special codes:  99 = Information missing | Weight \|___ ___ kg |  |
|  | Vital signs at admission (Day 0)  [A] Temperature (in Celsius)  [B] Respiratory rate  [C] Pulse rate  [D] SpO2  Special codes:  999 = Information missing | [A] Temperature ___ ___  [B] Respiratory rate ___ ___  [C] Pulse rate ___ ___ ___  [D] SpO2 ___ ___ ___ |  |
|  | Was the patient referred here from another facility? | Yes 1  No 2 |  |
|  | Name of the referring facility |  |  |
|  | LEVEL of the referring facility |  |  |
|  | Lab exams ordered at admission (Day 0)  Mark all that apply. | Blood culture A  Blood glucose B  Chest X-ray C  Erythrocyte sedimentation rate (ESR) D  Full blood cell count (FBC) E  Malaria parasite smear (MPS) or RDT F  Sputum test G  Stool analysis H  Throat swab I  Urine analysis J  Ultrasound K  COVID-19 L  Other (specify) X  None Z |  |
|  | Lab results |  |  |
|  | Initial/provisional diagnoses at admission (Day 0) |  |  |
|  | Treatments prescribed at admission (Day 0)  Mark all that apply. | Ampicillin A  Amoxcillin B  Antihelminthic C  Antihistamine D  Artesunate injectable E  Artemesinin combination therapy (ACT) F  Azithromycin G  Benzyl penicillin H  Ceftaxidime I  Ceftriaxone J  Cephalexin K  Chloramphenicol L  Clarithomycin M  Cotrimoxazole N  Cough syrup/tablets O  Dexamethasone P  Doxycycline Q  Gentamicin R  Intravenous fluid dextrose/saline S  Levofloxacin T  Metronidazole U  Nasogastric fluids/feeding V  Oral rehydration salts (ORS) W  Oxygen AA  Paracetamol BB  Paraldehyde injectable CC  Phenobarbitone injectable DD  Salbutamol EE  Saline drops FF  Vitamin tablets GG  Vitamin injection HH  Zinc sulphate tablets II  Other (*specify*) X  None Z |  |
| **Instructions:** In this next section, you will gather detailed information about the first day the patient was admitted at the facility. The patient was admitted on {pc1} so in the next section you will answer questions about {pc1}+1. Pay close attention to new symptoms, diagnoses, vital signs, and treatments. | | | |
|  | Are there case notes available for {pc1}+1? | Yes 1  No 2 | 1 🡪 PC19  2 🡪 PC23 |
|  | New/continuing symptoms noted on {pc1}+1  Mark all that apply. | **Respiratory**  Chest in-drawing R01  Cold R02  Cough R03  Crackles R04  Cyanosis/blue discoloration of the skin R05  Difficulty breathing R06  Fast breathing/Tachypnea R07  Grunting sound R08  Hypoxemia (low oxygen saturation) R09  Nasal flaring R10  Stridor (wheezing/grating sound) when  child is calm R11  Wheezing sound R12  Bradypnea R13  **Gastro/Feeding**  Diarrhoea F1  Unable to eat normally F2  Unable to drink normally F3  Vomiting F4  Nausea F5  Abdominal pain F6  **Neuro**  Convulsions N1  Drowsy or difficult to rouse N2  Pain (including headache and joint pain) N3  Restlessness or easily irritable N4  Unable to sleep normally N5  Unconscious N6  **General**  Fatigue or no energy G1  Fever G2  Shivering or chills G3  Dehydration G4  Other (*specify*) X  None (no symptoms recorded) Z |  |
|  | Vital signs on {pc1}+1  [A] Temperature (in Celsius)  [B] Respiratory rate  [C] Pulse rate  [D] SpO2  Special codes:  999 = Information missing | [A] Temperature ___ ___  [B] Respiratory rate ___ ___  [C] Pulse rate ___ ___ ___  [D] SpO2 ___ ___ ___ |  |
|  | New and updated diagnoses on {pc1}+1 |  |  |
|  | New and continuing treatments on {pc1}+1  Mark all that apply. | Ampicillin A  Amoxcillin B  Antihelminthic C  Antihistamine D  Artesunate injectable E  Artemesinin combination therapy (ACT) F  Azithromycin G  Benzyl penicillin H  Ceftaxidime I  Ceftriaxone J  Cephalexin K  Chloramphenicol L  Clarithomycin M  Cotrimoxazole N  Cough syrup/tablets O  Dexamethasone P  Doxycycline Q  Gentamicin R  Intravenous fluid dextrose/saline S  Levofloxacin T  Metronidazole U  Nasogastric fluids/feeding V  Oral rehydration salts (ORS) W  Oxygen AA  Paracetamol BB  Paraldehyde injectable CC  Phenobarbitone injectable DD  Salbutamol EE  Saline drops FF  Vitamin tablets GG  Vitamin injection HH  Zinc sulphate tablets II  Other (*specify*) X  None Z |  |
| **Instructions:** You have completed questions for {pc1}+1. In this next section, you will answer questions about the last day the child was at the facility before being discharged. You had previously noted that the child was discharged on {pc2}. | | | |
|  | Are there case notes available for {pc2}? | Yes 1  No 2 | 1 🡪 PC24  2 🡪 PC29 |
|  | New/continuing symptoms noted on {pc2}  Mark all that apply. | **Respiratory**  Chest in-drawing R01  Cold R02  Cough R03  Crackles R04  Cyanosis/blue discoloration of the skin R05  Difficulty breathing R06  Fast breathing/Tachypnea R07  Grunting sound R08  Hypoxemia (low oxygen saturation) R09  Nasal flaring R10  Stridor (wheezing/grating sound) when  child is calm R11  Wheezing sound R12  Bradypnea R13  **Gastro/Feeding**  Diarrhoea F1  Unable to eat normally F2  Unable to drink normally F3  Vomiting F4  Nausea F5  Abdominal pain F6  **Neuro**  Convulsions N1  Drowsy or difficult to rouse N2  Pain (including headache and joint pain) N3  Restlessness or easily irritable N4  Unable to sleep normally N5  Unconscious N6  **General**  Fatigue or no energy G1  Fever G2  Shivering or chills G3  Dehydration G4  Other (*specify*) X  None (no symptoms recorded) Z |  |
|  | Child’s weight on {pc2} (in grams)  Special codes:  99 = Information missing | Weight ___ ___ ___ ___ grams |  |
|  | Vital signs on {pc2}  [A] Temperature (in Celsius)  [B] Respiratory rate  [C] Pulse rate  [D] SpO2  Special codes:  999 = Information missing | [A] Temperature ___ ___  [B] Respiratory rate ___ ___  [C] Pulse rate ___ ___ ___  [D] SpO2 ___ ___ ___ |  |
|  | New and updated diagnoses on {pc2} |  |  |
|  | New and continuing treatments on {pc2}  Mark all that apply. | Ampicillin A  Amoxcillin B  Antihelminthic C  Antihistamine D  Artesunate injectable E  Artemesinin combination therapy (ACT) F  Azithromycin G  Benzyl penicillin H  Ceftaxidime I  Ceftriaxone J  Cephalexin K  Chloramphenicol L  Clarithomycin M  Cotrimoxazole N  Cough syrup/tablets O  Dexamethasone P  Doxycycline Q  Gentamicin R  Intravenous fluid dextrose/saline S  Levofloxacin T  Metronidazole U  Nasogastric fluids/feeding V  Oral rehydration salts (ORS) W  Oxygen AA  Paracetamol BB  Paraldehyde injectable CC  Phenobarbitone injectable DD  Salbutamol EE  Saline drops FF  Vitamin tablets GG  Vitamin injection HH  Zinc sulphate tablets II  Other (*specify*) X  None Z |  |
|  | Did the patient receive oxygen? | Yes 1  No 2 |  |
|  | If yes, how many days did the patient receive oxygen? |  |  |
|  | What was the average flow rate? |  |  |
|  | If there is any additional important information on this patient case that has not yet been captured, such as anything that may have affected the quality of the abstraction, record it here.  You may leave this blank if there are no additional comments. | --------------------------------------------------------- |  |
