# Supplementary material for: Hypoxemia detection and oxygen therapy practices in neonatal and pediatric wards across seven district and referral hospitals in Rwanda
Source: Front Pediatr. 2025 Mar 6;13:1526779. doi: 10.3389/fped.2025.1526779 (PMC11922911; doi:10.3389/fped.2025.1526779)
Supplement: Supplementary file 2 [file Table1.docx]

**Supplementary Annex B: Full list of diagnoses recorded for inpatients included in the study by month (N=3085)**

|  | **July 2021** | **August 2021** | **September 2021** | **Total** |
| --- | --- | --- | --- | --- |
| **Neonatal diagnoses on admission (< 1 month)**** | | | | |
| Neonatal Infection (Risk) | 149 (28.4) | 169 (31.0) | 149 (35.6) | 467 (31.4) |
| Prematurity | 90 (17.1) | 113 (20.7) | 74 (17.7) | 277 (18.6) |
| Respiratory Distress Syndrome | 69 (13.1) | 66 (12.1) | 44 (10.5) | 179 (12.0) |
| Birth asphyxia | 72 (13.7) | 49 (9.0) | 39 (9.3) | 160 (10.8) |
| Jaundice | 21 (4.0) | 32 (5.9) | 19 (4.5) | 72 (4.8) |
| Hypoglycemia | 28 (5.3) | 16 (2.9) | 16 (3.8) | 60 (4.0) |
| Septicemia | 17 (3.2) | 18 (3.3) | 17 (4.1) | 52 (3.5) |
| Encephalopathies | 15 (2.9) | 16 (2.9) | 18 (4.3) | 49 (3.3) |
| Respiratory Tract Infection | 13 (2.5) | 18 (3.3) | 8 (1.9) | 39 (2.6) |
| Pneumonia | 9 (1.7) | 10 (1.8) | 4 (1.0) | 23 (1.5) |
| Congenital Malformations | 9 (1.7) | 7 (1.3) | 6 (1.4) | 22 (1.5) |
| Aspiration | 9 (1.7) | 6 (1.1) | 4 (1.0) | 19 (1.3) |
| Abdominal / Gastrointestinal | 3 (0.6) | 7 (1.3) | 8 (1.9) | 18 (1.2) |
| Seizures / Convulsions | 3 (0.6) | 7 (1.3) | 4 (1.0) | 14 (0.9) |
| Anemia | 5 (1.0) | 1 (0.2) | 2 (0.5) | 8 (0.5) |
| Apnea | 6 (1.1) | 0 (0.0) | 1 (0.2) | 7 (0.5) |
| Malaria | 2 (0.4) | 2 (0.4) | 2 (0.5) | 6 (0.4) |
| COVID-19 | 1 (0.2) | 3 (0.6) | 0 (0.0) | 4 (0.3) |
| Malnutrition | 1 (0.2) | 1 (0.2) | 1 (0.2) | 3 (0.2) |
| Sickle Cell Anemia | 1 (0.2) | 2 (0.4) | 0 (0.0) | 3 (0.2) |
| Urinary Tract Infection | 1 (0.2) | 1 (0.2) | 0 (0.0) | 2 (0.1) |
| Typhoid | 1 (0.2) | 0 (0.0) | 1 (0.2) | 2 (0.1) |
| Tuberculosis | 0 (0.0) | 1 (0.2) | 0 (0.0) | 1 (0.1) |
| Diarrhea | 0 (0.0) | 0 (0.0) | 1 (0.2) | 1 (0.1) |
| **Pediatric diagnoses on admission (1 month – 14 years)**** | | | | |
| Malaria | 88 (22.1) | 125 (20.8) | 67 (15.9) | 280 (19.7) |
| Pneumonia | 87 (21.8) | 101 (16.8) | 71 (16.9) | 259 (18.2) |
| Abdominal / Gastrointestinal | 47 (11.8) | 103 (17.1) | 87 (20.7) | 237 (16.7) |
| Diarrhea | 39 (9.8) | 80 (13.3) | 64 (15.2) | 183 (12.9) |
| Septicemia | 25 (6.3) | 41 (6.8) | 25 (5.9) | 91 (6.4) |
| Respiratory Tract Infection | 31 (7.8) | 28 (4.7) | 27 (6.4) | 86 (6.1) |
| Seizures / Convulsions | 19 (4.8) | 28 (4.7) | 30 (7.1) | 77 (5.4) |
| COVID-19 | 13 (3.3) | 24 (4) | 9 (2.1) | 46 (3.2) |
| Urinary Tract Infection | 12 (3) | 15 (2.5) | 10 (2.4) | 37 (2.6) |
| Malnutrition | 8 (2) | 16 (2.7) | 6 (1.4) | 30 (2.1) |
| Asthma | 10 (2.5) | 9 (1.5) | 5 (1.2) | 24 (1.7) |
| Typhoid | 4 (1) | 8 (1.3) | 5 (1.2) | 17 (1.2) |
| Meningitis | 4 (1) | 6 (1) | 5 (1.2) | 15 (1.1) |
| Sickle Cell Anemia | 7 (1.8) | 6 (1) | 2 (0.5) | 15 (1.1) |
| Encephalopathies | 2 (0.5) | 6 (1) | 3 (0.7) | 11 (0.8) |
| Trauma | 2 (0.5) | 3 (0.5) | 3 (0.7) | 8 (0.6) |
| Tuberculosis | 1 (0.3) | 2 (0.3) | (0) | 3 (0.2) |
| Otis Media | (0) | (0) | 2 (0.5) | 2 (0.1) |
